# Calibration of a density-based model of urban morphogenesis

**DOI:** 10.1371/journal.pone.0203516

**Published:** 2018-09-06

**Authors:** Juste Raimbault

**Affiliations:** 1 UMR CNRS 8504 Géographie-cités, Paris, France; 2 UMR-T 9403 IFSTTAR LVMT, Champs-sur-Marne, France; University College London, UNITED KINGDOM

## Abstract

We study a stochastic model of urban growth generating spatial distributions of population densities at an intermediate mesoscopic scale. The model is based on the antagonist interplay between the two opposite abstract processes of aggregation (preferential attachment) and diffusion (urban sprawl). Introducing indicators to quantify urban form, the model is first statistically validated and intensively explored to understand its complex behavior across the parameter space. We then compute real morphological indicators on local areas of size 50km covering all European Union, and show that the model can reproduce most of these existing urban morphologies. It implies that the morphological dimension of urban growth processes at this scale are sufficiently captured by the two abstract processes of aggregation and diffusion.

## Introduction

The study of urban growth, and more particularly its quantification, is more than ever a crucial issue in a context where most of the world population live in cities which expansion has significant environmental impacts [1] and that have therefore to ensure an increased sustainability and resilience to climate change. The understanding of drivers for urban growth can lead to better integrated policies. This remains however a question far from being solved in the diverse related disciplines: urban systems are complex socio-technical systems that can be studied from a large variety of viewpoints. [2] advocates in that sense for the construction of a dedicated science defined by its objects of study more than the methods used, what would allow easier coupling of approaches and therefore urban growth models taking into account heterogeneous processes.

The processes that a model can grasp are also linked to the choice of the scale of study. At a macroscopic scale, models of growth in system of cities are mainly the concern of economics and geography. [3] shows that in first approximation, the Gibrat’s model postulating random growth rates that do not depend on city size, produces the well-know Zipf’s law. This rank-size law is a typical stylized fact witnessing hierarchy in systems of cities. It was however shown empirically that systematic deviations to this law exist [4], and that spatial interactions may contribute to these. Models integrating spatial interactions include for example the one proposed by [5]. They introduce a growth model in which these interactions, that depend on distance and the geography, play a significant role in growth rates. More recently, [6] has extended this model by taking into account innovation waves between cities as an additional driver of growth. The interplay between space, economics and population growth is studied by the Marius model [7] in the case of the former Soviet Union, on which model performance is considerably improved in comparison to models without interactions.

At smaller scales, that can be understood as microscopic or mesoscopic depending on the resolution and extent of models, agents of models fundamentally differ. Space is generally taken into account in a finer way, through neighborhood effects for example. For example, [8] propose a microscopic model of urban growth, with the purpose to replace non-interpretable physical mechanisms with agent mechanisms, including interactions forces and mobility choices. Local correlations are used by [9], which develop the model introduced in [10], in order to modulate growth patterns to approximate real configurations. The family of cellular automatons (CA) models of urban growth [11] also offers numerous examples. [12] introduces a generic framework for CA with multiple dimensions for land use, based on local evolution rules. A model with simpler states (occupied or not) but taking into account global constraints is studied by [13]. The Sleuth model, initially introduced by [14] for the San Francisco Bay area, and for which an overview of diverse applications is given in [15], was calibrated on metropolitan areas all over the world, providing comparative measures through the calibrated parameters.

Closely related to CA models but not exactly similar are urban morphogenesis models, which aim to simulate the growth of urban form from autonomous rules. [16] suggests that the fractal nature of cities is closely linked to the emergence of the form from the microscopic socio-economic interactions, namely urban morphogenesis. [17] develop a morphogenesis model for urban roads alone, with growth rules based on geometrical considerations. These are shown sufficient to produce a broad range of patterns resembling existing ones. Similarly, [18] couple a CA with an evolving network to reproduce stylized urban form, from concentrated monocentric cities to sprawled suburbs. The diffusion-limited-aggregation model (DLA), imported from physics, and which was first studied for cities by [19], can also be seen as a morphogenesis model. The particularity of morphogenesis models, in comparison to cellular automatons, is the crucial role of the urban form in their evolution rules, and for some of the urban functions, such as in [20]. These kind of models, that sometimes can be classified as CA, have generally the particularity of being parsimonious in their structure. Similar models have also been studied in biology for the diffusion of population as for example [21]. Morphogenesis is by essence interdisciplinary, and [22] have recently applied ideas from medical science and an CA urban growth model to elaborate strategies for urban growth management.

We study in this paper a morphogenesis model at the mesoscopic scale. The model aims at being simplistic in its rules and variables, but also being accurate in the reproduction of existing patterns. The underlying question is to explore the performance of simple mechanisms in reproducing complex urban patterns. We consider abstract processes, namely aggregation and diffusion, candidates as partly explanatory drivers of urban growth. These are based on population only and will be detailed in model rationale below. An important aspect we introduce is the quantitative measure of urban form, based on a combination of morphological indicators, to quantify and compare model outputs and real urban patterns. Our contribution is significant on several points: (i) we compute local morphological characteristics on a large spatial extent (full European Union); (ii) we give significant insights into model behavior through extensive exploration of the parameter space; (iii) we show through calibration that the model is able to reproduce most of existing urban forms across Europe, and that these abstract processes are sufficient to explain urban form.

The rest of this paper is organized as follows: we first describe formally the model and the morphological indicators. We then detail values of morphological measures on real data, study the behavior of the model by exploring its parameter space and through a semi-analytical approach to a simplified case, and we finally describe results of model calibration.

## Material and methods

### Urban growth model

#### Rationale

Our model is based on widely accepted ideas of diffusion-aggregation processes for urban processes. The combination of attraction forces with repulsion, due for example to congestion, already yield a complex outcome that has been shown under some simplifying assumptions to be representative of urban growth processes. A model capturing these processes was introduced by [23], as a cell-based variation of the DLA model [19]. Indeed, the tension between antagonist aggregation and sprawl mechanisms may be an important process in urban morphogenesis. [24] opposes centrifugal forces with centripetal forces in the equilibrium view of urban spatial systems, what is easily transferable to non-equilibrium systems in the framework of self-organized complexity: a urban structure is a far-from-equilibrium system that has been driven to this point by these opposite forces. For example, concrete dispersion forces are congestion or the search for low density by residents, whereas aggregation forces can be the presence of amenities, places of interest, or increased potentialities of social interactions [25]. Empirical evidence of a combination of diffusion and coalescence, which can be in our case interpreted as aggregation, has been furthermore shown by [26]. [27] also combine percolation processes with diffusion of migrant agents to simulate urbanization at the scale of a country.

The two contradictory processes of urban concentration and urban sprawl are captured by the model, what allows to reproduce with a good precision a large number of existing morphologies. We can expect aggregation mechanisms such as preferential attachment to be good candidates in urban growth explanation, as it was shown that the Simon model based on these generates power-laws typical of urban systems such as scaling laws [28]. The question at which scale it is possible and relevant to define and try to simulate urban form is rather open, and will in fact depend on which issues are being tackled. Working in a typical setting of morphogenesis, the processes considered are local and our model must have a resolution at the micro-level. We however want to quantify urban form on consistent urban entities, and will work therefore on spatial extents of order 50-100km. We sum up these two aspects by stating that the model is at the *mesoscopic scale*. In comparison, the microscopic scale would correspond to the intra-urban scale and ranges under 10km, whereas the macroscopic scale would be the scale of the system of cities with extents of size 500-1000km.

#### Formalization

We formalize now the model and its parameters. The world is a square grid of width *N*, in which each cell is characterized by its population ${\left(P_{i}\left(t\right)\right)}_{1\leq i\leq N^{2}}$. We consider that the grid initially empty, i.e. *P*_*i*_(0) = 0, but the model can be easily generalized to any initial population distribution. The population distribution is updated in an iterative way. At each time step,

1. Total population is increased by a fixed number *N*_*G*_ (growth rate). Each population unit is attributed independently to a cell following a preferential attachment such that
  $$\begin{array}{c} \text{P}\;[P_{i}\left(t+1\right)=P_{i}\left(t\right)+1|P\left(t+1\right)=P\left(t\right)+1]=\frac{{\left(P_{i}\left(t\right)/P\left(t\right)\right)}^{\alpha }}{\sum {\left(P_{i}\left(t\right)/P\left(t\right)\right)}^{\alpha }} \end{array}$$ (1)
  The attribution is uniformly drawn when all cell populations are equal to 0.
2. A fraction *β* of population is diffused to cell neighborhood (8 closest neighbors receiving each the same fraction of the diffused population). This operation is repeated *n*_*d*_ times.

The model stops when total population reaches a fixed parameter *P*_*m*_. To avoid side effects such as reflecting diffusion waves, border cells diffuse their due proportion outside of the world, implying that the total population at time *t* is strictly smaller than *N*_*G*_ ⋅ *t*.

We summarize model parameters in Table 1, giving the associated processes and values ranges we use in the simulations. The total population of the area *P*_*m*_ is exogenous, in the sense that it is supposed to depend on macro-scale growth patterns on long times. Growth rate *N*_*G*_ captures both endogenous growth rate and migration balance within the area. The aggregation rate *α* sets the differences in attraction between cells, what can be understood as an abstract attraction coefficient following a scaling law of population. Finally, the two diffusion parameters are complementary since diffusing with strength *n*_*d*_ ⋅ *β* is different of diffusing *n*_*d*_ times with strength *β*, the later giving flatter configurations. Boundaries for parameters are obtained through successive experimentations as detailed below.

**Table 1:** Summary of model parameters.

Parameter	Notation	Process	Range
Total population	*P*_*m*_	Macro-scale growth	[1*e*4, 1*e*6]
Growth rate	*N*_*G*_	Meso-scale growth	[500, 30000]
Aggregation strength	*α*	Aggregation	[0.1, 4]
Diffusion strength	*β*	Diffusion	[0, 0.5]
Diffusion steps	*n*_*d*_	Diffusion	{1, … , 5}

#### Quantifying urban form

As our model is only density-based, we propose to quantify its outputs through spatial morphology, i.e. properties of the spatial distribution of density. At the scale chosen, these will be expected to capture various functional properties of the urban landscape. [29] study the form of European cities using a simple measure of density slopes from the center to the periphery. We need however quantities having a certain level of robustness and invariance. For example, two polycentric cities should be classified as morphologically close whereas a direct comparison of distributions (with the Earth Mover Distance for example) could give a very high distance between configurations depending on the position of centers. The use of fractal indices is an other possibility to quantify urban form suggested by [30]. Other more original indices can be proposed, such as by [31] which use the variations of trajectories for routes going through a city to establish a classification and show that it is strongly correlated with socio-economic variables.

We choose to refer to the literature in urban morphology which proposes an extensive set of indicators to describe urban form [32]. The number of dimensions can be reduced using principal component analysis to obtain a robust description with a few number of independent indicators [33]. Note that we consider here indicators on population density only, and that more elaborated considerations on urban form include for example the distribution of economic opportunities and the combination of these two fields through accessibility measures. For the choice of indicators, we follow the analysis done in [34] in which a morphological typology of large European cities is obtained.

We give now the formal definition of morphological indicators. We write *M* = *N*^2^ the number of cells, *d*_*ij*_ the distance between cells *i*, *j*, and $P=\sum_{i=1}^{N}P_{i}$ total population. We quantify the urban form using:

1. Rank-size slope *γ*, expressing the degree of hierarchy in the distribution, computed by fitting with Ordinary Least Squares a power law distribution by $\ln(P_{\tilde{i}}/P_{0})∼k+\gamma \cdot \ln(\tilde{i}/i_{0})$ where $\tilde{i}$ are the indexes of the distribution sorted in decreasing order. It is always negative, and values close to zero correspond to a flat distribution.
2. Entropy of the distribution, that captures how uniform the distribution is:
  $$\begin{array}{c} E=\sum_{i=1}^{M}\frac{P_{i}}{P}\cdot \ln\frac{P_{i}}{P} \end{array}$$ (2)
  E=0 means that all the population is in one cell whereas E=1 means that the population is uniformly distributed.
3. Spatial-autocorrelation given by Moran index, with simple spatial weights given by *w*_*ij*_ = 1/*d*_*ij*_
  $$\begin{array}{c} I=M\cdot \frac{\sum_{i\neq j}w_{ij}(P_{i}-\bar{P})\cdot (P_{j}-\bar{P})}{\sum_{i\neq j}w_{ij}\sum_{i}{\left(P_{i}-\bar{P}\right)}^{2}} \end{array}$$ (3)
  Its theoretical boundaries are -1 and 1, and positive values imply aggregation spots (“density centers”), negative values strong local variations whereas *I* = 0 corresponds to totally random population values.
4. Average distance between individuals [35], which captures a spatial dispersion of population and quantifies a level of acentrism (distance to a monocentric model):
  $$\begin{array}{c} \bar{d}=\frac{1}{d_{M}}\cdot \sum_{i<j}\frac{P_{i}P_{j}}{P^{2}}\cdot d_{ij} \end{array}$$ (4)
  where *d*_*M*_ is a normalisation constant taken as the length of the diagonal of the world in our case.

The first two indices are not spatial, and are completed by the last two that take space into account. Following [33], the effective dimension of the urban form justifies the use of all.

### Real data

We compute the morphological measures given above on real urban density data, using the population density grid of the European Union at 100m resolution provided openly by Eurostat [36]. The choice of the resolution, the spatial range, and the shape of the window on which indicators are computed, is made according to the thematic specifications of the model. We consider 50km wide square windows to be in accordance with the expected spatial range of one model instance. As it also does not make sense to have a too detailed resolution because of data quality, we take *N* = 100 and aggregate the initial raster data at a 500m resolution to meet this size on real windows. According to [37] which details the construction of the dataset, good results were obtained after validation for seven countries on samples with a grid of resolution 1km. We are thus closer of this resolution with a resolution of 500m. This moderates the biais highlighted for example by [38].

To have a rather continuous distribution of indicators in space, we overlap windows by setting an offset of 10km between each, what also somehow rules out the question of window shape bias by introducing a continuity of values. We tested the sensitivity to window size by computing also the values with 30km and 100km window sizes and obtained rather similar spatial distributions, and also strong correlations between the fields and their smoothing at a finer resolution, as detailed in S3 Text.

**Fig 1:**
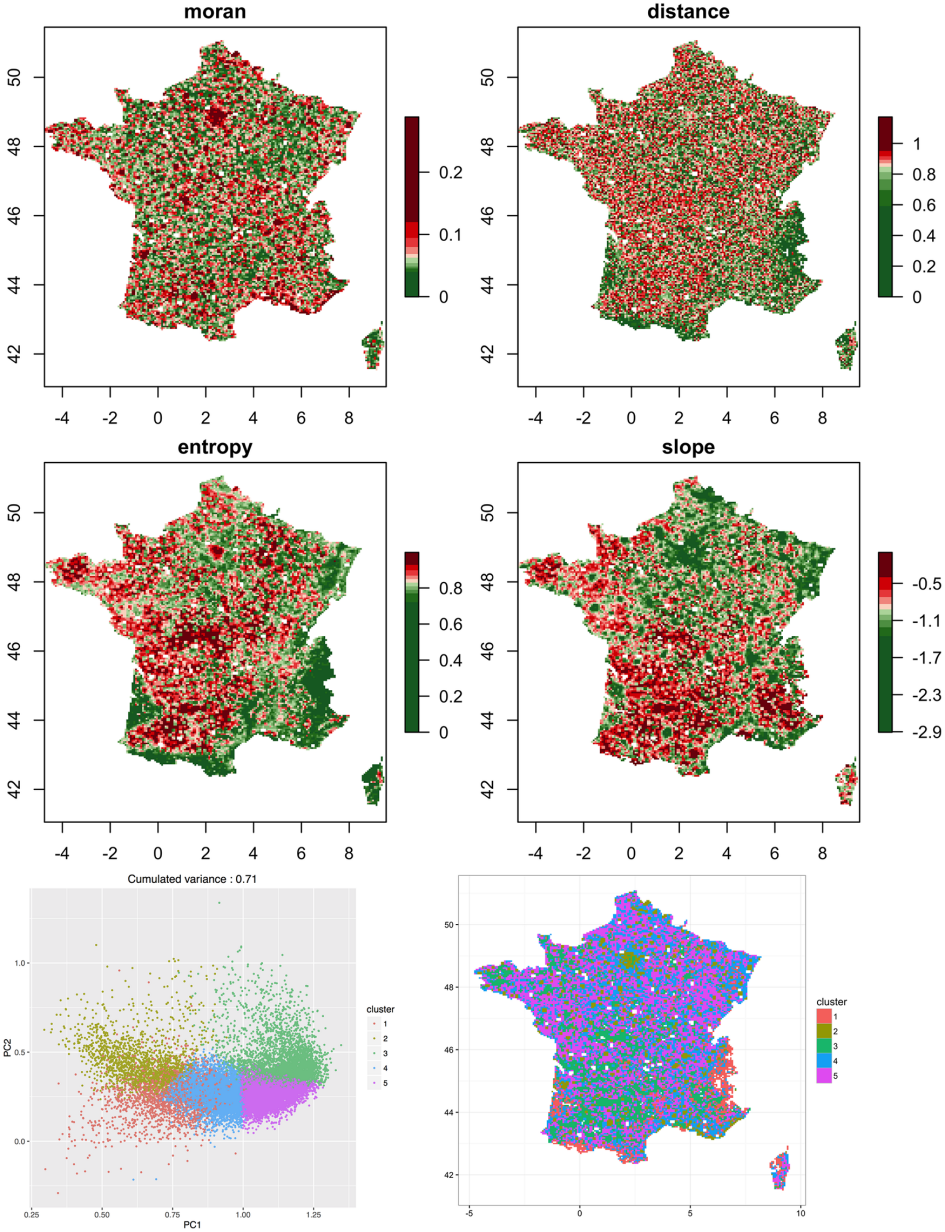
Empirical values of morphological indicators. *(Top four maps)* Spatial distribution of the morphological indicators for France. Scale color discretization is done using quantiles to ease map readability. *Bottom Left* Projection of morphological values on the two first components on a Principal Component analysis. Color gives the cluster in an unsupervised classification (see text). *Bottom right* Spatial distribution of clusters.

We show in Fig 1 maps giving values of indicators for France only to ease maps readability. The first striking feature is the diversity of morphological patterns across the full territory. The auto-correlation is naturally high in Metropolitan areas, with the Parisian surroundings clearly detached. When looking at other indicators, it is interesting to observe regional regimes: rural areas have much less hierarchy in the South than in the North, whereas the average distance is rather uniformly distributed except for mountainous areas. Regions with a very high entropy are observed in the Center and South-West. To have a better insight into morphological regimes, we use unsupervised classification with a simple k-means algorithm (with *b* = 100 repetitions of the algorithm in order to be robust to stochasticity), for which the number of clusters *k* = 5 witnesses a transition in inter-cluster variance. The separation between classes is plotted in Fig 1, bottom-left panel, where we show measures projected on the two first components of a principal component analysis (explaining 71% of variance). The map of morphological classes confirms a North-South opposition in a background rural regime (clear green against blue), the existence of mountainous (red) and metropolitan (dark green) regimes. Such a variety of settlements forms will be the target for the model.

## Results

### Generation of urban patterns

#### Implementation

The model is implemented both in NetLogo [39] for exploration and visualization purposes, and in Scala for performance reasons and easy integration into the model exploration software OpenMole [40], which allows a transparent access to high performance computing environments and provides methods for model exploration and calibration. Computation of indicator values on geographical data is done in R using the raster package [41], which uses fast Fourier transform for spatial convolution. This method lowers the computational complexity from a *O*(*N*^4^) to a *O*(*N*^2^ log^2^ *N*), what is already significant with the sizes we consider. This method was implemented in the Scala model and included in a NetLogo extension developed on purpose.

Source code and results are available on the open repository of the project at https://github.com/JusteRaimbault/Density. Raw datasets for real indicator values and simulation results are available on Dataverse at http://dx.doi.org/10.7910/DVN/WSUSBA. The computational protocol of this study is openly archived for reproducibility at https://dx.doi.org/10.17504/protocols.io.repd3dn.

**Fig 2:**
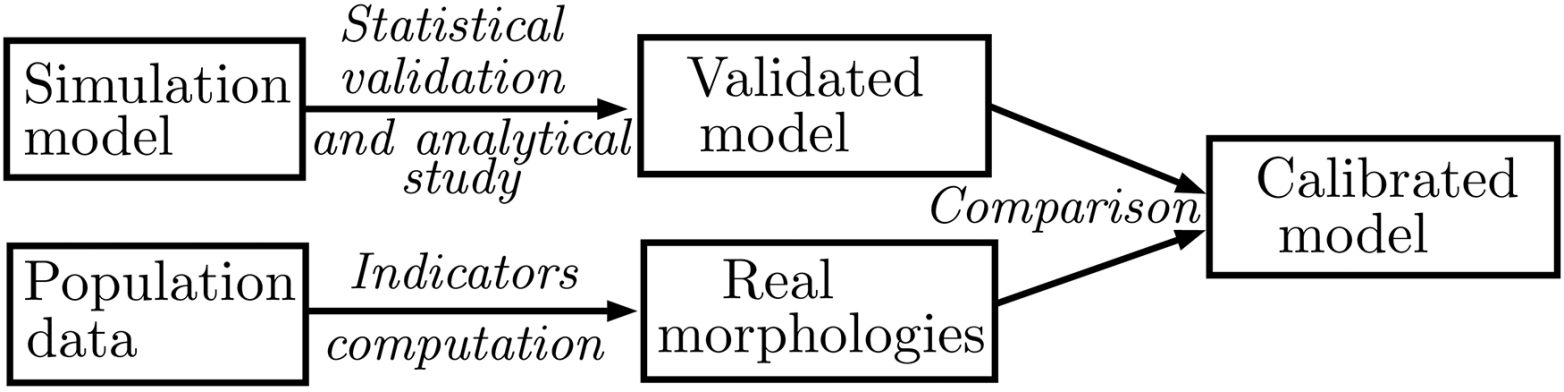
Flowchart summarizing the main steps of the approach to obtain a calibrated model.

A flowchart summarizing our method is shown in Fig 2. Its main points are one the one hand, the validation and exploration of the simulation model, and on the other hand the computation of indicators on real data, in order to finally obtain a calibrated model.

#### Generated shapes

The model has a relatively small number of parameters but is able to generate a large variety of shapes, extending beyond existing forms. In particular, its dynamical nature allows through the interplay between *P*_*m*_ and *N*_*G*_ parameters to choose between final configurations that can be non-stationary or semi-stationary, whereas the interplay between *α* and *β* modulates the sprawl and the compactness of forms. We run the model for parameters varying in ranges given in Table 1, for a world size *N* = 100.

Since relative values of *P*_*m*_ and *N*_*G*_ have a stronger influence on the forms obtained than their values in absolute, and we thus set *P*_*m*_ to obtain territories containing at most 1 million inhabitants, what is a strong but not extreme density (for comparison, the Parisian region concentrates around 8 millions inhabitants on an area of a similar size). Values of *N*_*G*_ vary considerably to cover a large number of possible dynamical regimes. Values of *α* and *β* have been obtained through successive experimentations. We take therefore the following boundaries for parameters: *α* ∈ [0.1, 4], *β* ∈ [0, 0.5], *n*_*d*_ ∈ {1, … , 5}, *N*_*G*_ ∈ [500, 30000], *P*_*m*_ ∈ [1*e*4, 1*e*6].

**Fig 3:**
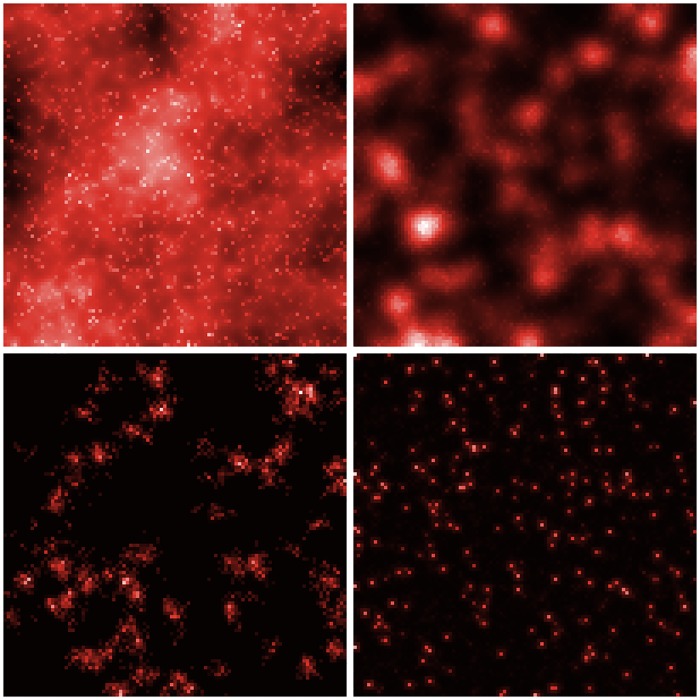
Example of the variety of generated urban shapes. *(Top left)* Very diffuse urban configuration, *α* = 0.4, *β* = 0.05, *n*_*d*_ = 2, *N*_*G*_ = 76, *P*_*m*_ = 75620; *(Top Right)* Semi-stationary polycentric urban configuration, *α* = 1.4, *β* = 0.047, *n*_*d*_ = 2, *N*_*G*_ = 274, *P*_*m*_ = 53977; *(Bottom Left)* Intermediate settlements (peri-urban or densely populated rural area), *α* = 0.4, *β* = 0.006, *n*_*d*_ = 1, *N*_*G*_ = 25, *P*_*m*_ = 4400; *(Bottom Right)* Rural area, *α* = 1.6, *β* = 0.006, *n*_*d*_ = 1, *N*_*G*_ = 268, *P*_*m*_ = 76376. Population values can be counter-intuitive here, as the final form of the density is determined by *P*_*m*_/*N*_*G*_ more than total population only, and our interpretation here is based on renormalized density. A targeted calibration out of the scope of this work could produce more realistic values through conditional calibration.

Fig 3 shows examples of the variety of generated shapes for different parameter values, with corresponding interpretations. The four very different shapes can be obtained sometimes by varying a single parameter: transiting from a peri-urban area to a rural area implies an increased aggregation at the same level of diffusion. Note that the model is density driven, and that the parameter *P*_*m*_/*N*_*G*_ is what really influences the dynamics: the values of *P*_*m*_ do in some cases not directly correspond to the interpretations we made (for the rural in particular) that are done on densities. A rescaling keeps the settlement form and solves this issue.

It appears that the dynamical nature of the model allows through the combination of parameters *P*_*m*_ and *N*_*G*_ to choose between configurations that can be non-stationary or semi-stationary, whereas the interaction between *α* and *β* modulates the sprawl and the compact character of forms.

These examples show the potentiality of the model to produce diverse shapes. We need then to systematically tackle its stochasticity and explore its parameter space.

### Model behavior

In the study of such a computational model of simulation, the lack of analytical tractability must be compensated by an extensive knowledge of model behavior in the parameter space [42]. This type of approach is typical of what [43] calls the *Computational shift in modern science*: knowledge is less extracted through analytical exact resolution than through intensive computational experiments, even for “simple” models such as the one we study.

#### Convergence

First of all we assess the behavior of the model regarding stochasticity and the convergence of the statistics of its output indicators on stochastic repetitions. We run the model for a sparse grid of the parameter space consisting of 81 points, with 100 repetitions for each point. Corresponding histograms are given in S1 Text. Indicators show good convergence properties: most of indicators are easily statistically discernable across parameter points: for example the Moran index, which is among the most dispersed, has a spread between 0 and 0.1 between parameters but a maximal variability of 0.01 between replications.

We use this experiment to determine a reasonable number of repetitions needed in larger experiments. For each point, we estimate the Sharpe ratios for each indicators, i.e. the average of the distribution normalized by its standard deviation. The more variable indicator is Moran with a minimal Sharpe ratio of 0.93, but for which the first quartile is at 6.89. Other indicators have very high minimal values, all above 2. Its means than confidence intervals large as 1.5 ⋅ *σ* are enough to differentiate between two different configurations. In the case of a gaussian distribution, we know that the size of the 95% confidence around the average is given by $2\cdot \sigma \cdot 1.96/\sqrt{n}$, what gives 1.26 ⋅ *σ* for *n* = 10. We run therefore this number of repetitions for each parameter point in the following, what is highly enough to have statistically significant differences between average as shown above. In the following, when referring to indicator values for the simulated model, we consider the ensemble averages on these stochastic runs.

#### Sensitivity analysis

We sample the Parameter space using a Latin Hypercube Sampling, with parameters varying in ranges given above. This type of cribbing is a good compromise to have a reasonable sampling without being subject to the dimensionality curse within normal computation capabilities. We sample around 80000 parameters points, with 10 repetitions each. Full plots of model behavior as a function of parameters are given in S1 Text. As we explored the full parameter range that we fixed for the model, this study of model behavior is equivalent to a sensitivity analysis. To understand a particular regime of interaction between aggregation and diffusion, one must refer to the corresponding range within the full model behavior. We detail now some regimes with interesting features.

**Fig 4:**
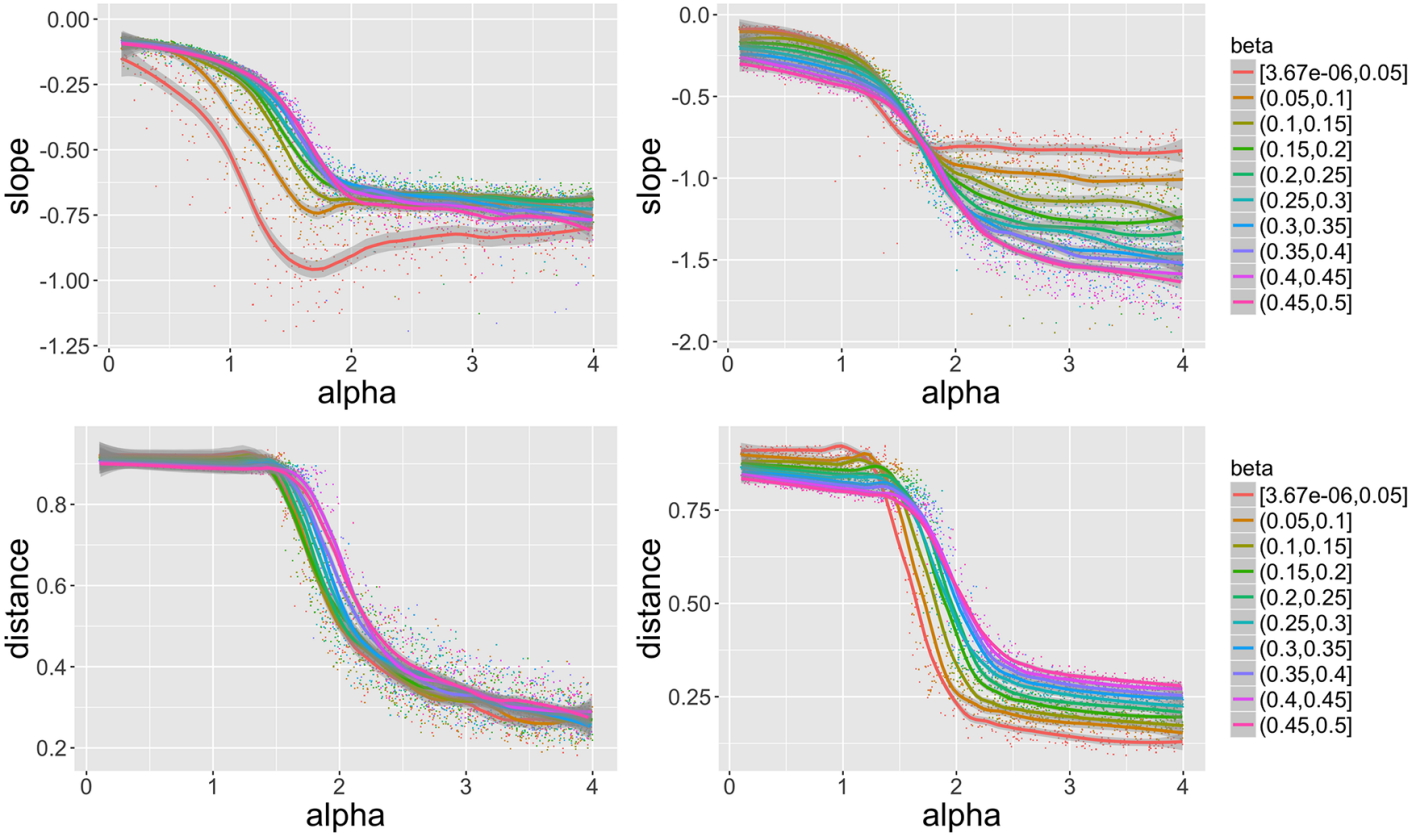
Behavior of indicators. Slope *γ* (top row) and average distance $\bar{d}$ (bottom row) as a function of *α*, for different bins for *β* given by curve color, for particular values *n*_*d*_ = 1, *P*_*m*_/*N*_*G*_ ∈ [13, 26] (left column) and *n*_*d*_ = 4, *P*_*m*_/*N*_*G*_ ∈ [41, 78] (right column). We observe in each case a transition as a function of *α*, which properties are influenced by other parameters. For low values of *P*_*m*_/*N*_*G*_ and of *β* emerges a counter-intuitive non-monotony.

We show in Fig 4 some particularly interesting behavior for slope *γ* and average distance $\bar{d}$. First of all, the overall qualitative behavior depending on aggregation strength, namely that lower alpha gives less hierarchical and more spread configurations, confirms the expected intuitive behavior.

The effect of diffusion strength *β* is more difficult to grasp: the effect is inverted for slope between high and low growth rates but not for distance, that shows an inversion when *α* varies. In the low *N*_*G*_ case, low diffusion creates more sprawled configuration when aggregation is low, but less sprawled when aggregation is high. Furthermore, all indicators show a more or less smooth transition around *α* ≃ 1.5. The slope stabilizes over certain values, meaning that the hierarchy cannot be forced more and indeed depends of the diffusion value, at least for low *N*_*G*_ (right column). In general, higher values for *P*_*m*_/*N*_*G*_ increase the effect of diffusion what could have been expected.

The existence of a minimum for the slope at *n*_*d*_ = 1, *P*_*m*_/*N*_*G*_ ∈ [13, 26] and lowest *β* is unexpected and witnesses a complex interplay between aggregation and diffusion. The emergence of this “optimal” regime is associated with shifts of the transition points in other cases: for example, lowest diffusion values imply a transition beginning at lower values of *α* for the average distance. This exploration confirms that complex behavior, in the sense of unpredictable emerging forms, occurs in the model: one cannot predict in advance the final form given some parameter values, without referring to the full exploration of which we give an overview here.

### Semi-analytical analysis

Our model can be understood as a type of reaction-diffusion model, that have been widely used in other fields such as biology: similar processes were used for example by Turing in its seminal paper on morphogenesis [44]. An other way to formulate the model typical to these approaches is by using partial differential equations (PDE). In the case of a firm growth model, which is a generalization of the Simon model with an arbitrary form of the attachment function, [45] show that a PDE and its general solution can be derived.

We propose to gain insights into long-time dynamics by studying our model from this point of view on a simplified case. We consider the system in one dimension, such that *x* ∈ [0;1] with 1/*δx* cells of size *δx*. A time step is given by *δt*. Each cell is characterized by its population as a random variable *P*(*x*, *t*). We work on their expected values p(x,t)=E[P(x,t)], and assume that *n*_*d*_ = 1. As developed in S2 Text, we show that this simplified process verifies the following PDE:

$$\begin{array}{c} \delta t\cdot \frac{\partial p}{\partial t}=\frac{N_{G}\cdot p^{\alpha }}{P_{\alpha }\left(t\right)}+\frac{\alpha \beta \left(\alpha -1\right)\delta x^{2}}{2}\cdot \frac{N_{G}\cdot p^{\alpha -2}}{P_{\alpha }\left(t\right)}\cdot {\left(\frac{\partial p}{\partial x}\right)}^{2}+\frac{\beta \delta x^{2}}{2}\cdot \frac{\partial^{2}p}{\partial x^{2}}\cdot [1+\alpha \frac{N_{G}p^{\alpha -1}}{P_{\alpha (t)}}] \end{array}$$ (5)

where *P*_*α*_(*t*) = ∫_*x*_ *p*(*x*, *t*)^*α*^ *dx*. This non-linear equation can not be solved analytically, the presence of integral terms putting it out of standard methods, and numerical resolution must be used [46]. It is important to note that the simplified model can be expressed by a PDE analog to reaction-diffusion equations, such as the one partially solved for a simpler model in [21].

We show in S2 Text that because of the boundaries conditions, density (proportion of population) converges towards a stationary solution at long times, going through intermediate states in which the solution is partially stabilized, in the sense that its evolution speed becomes rather slow. These “semi-stationary” states are the ones used in two dimensions along with the dynamical ones. This study confirms that the variety of shapes obtained through the model is permitted both by the interplay of aggregation and diffusion as the equation couples them, but also by the values of *P*_*m*_/*N*_*G*_ that allow to set the convergence level. Indeed, the sensitivity of the stationary solution to parameters is very low compared to the shape of the world, and using the model in a stationary mode would make no sense in our case.

**Fig 5:**
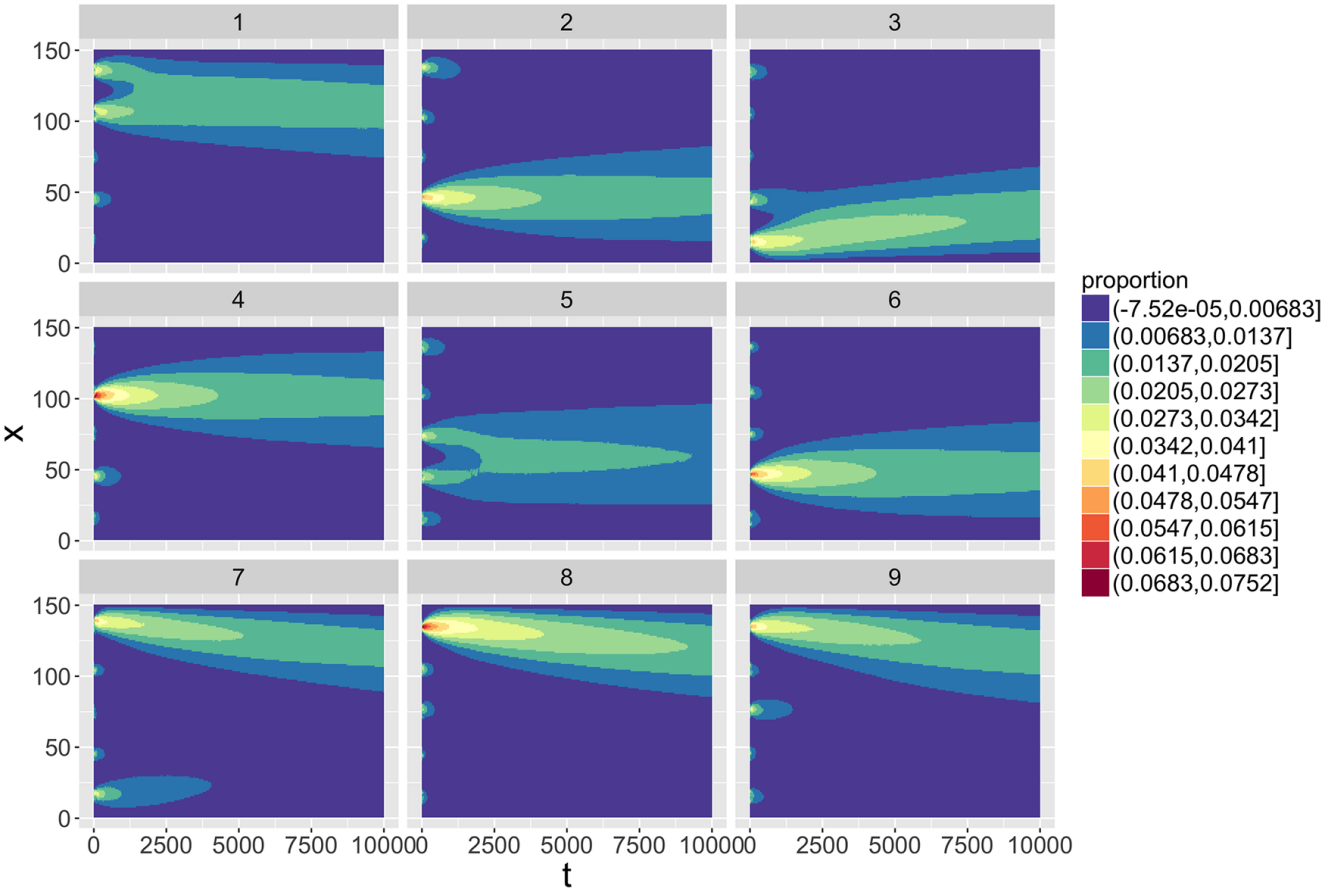
Randomness and frozen accidents. We show nine random realizations of the one dimensional system with similar initial conditions, namely five equidistant equally populated initial cells. Parameters are *α* = 1.4, *β* = 0.1, *N*_*G*_ = 10. Each plot shows time against space, color level giving the proportion of population in each cell.

Finally, we use this toy case to demonstrate the importance of bifurcations in model dynamics. More precisely, we show that path-dependence is crucial for the final form. As illustrated in Fig 5, we use an initial condition making the first choices of population attribution ambiguous, corresponding to five equidistant equally populated cells. This produces very different trajectories, as generally one of the spots will finally dominate the others, but is totally random, witnessing dramatic bifurcations in the system at initial times. This aspect is typically expected in urban systems, since very precise characteristics will be included in the determinants of localization at the initial moments of system genesis: the existence of a very local resource, or the strategic advantage of the site, will determine on long times the local territorial form. This confirms the importance of robust indicators described before.

### Model calibration

We finally turn to the the calibration of the model, that is done on the morphological indicators as calibration objectives. As a single calibration for each real cell is computationally out of reach, we use the previous model exploration and superpose the point clouds with real indicator values. Full scatterplots of all indicators against each other, for simulated and real configurations, are given in S1 Text. We find that the real point cloud is mostly contained within the simulated, which extends on significantly larger areas of the indicator space. It means that for a large majority of real configurations, there exist model parameters producing in average exactly the same morphological configuration. The highest discrepancy is for the distance indicator, the model failing to reproduce configuration with a high average distance, a low Moran and an intermediate hierarchy. These could for example correspond to polycentric configurations with many consequent centers.

**Fig 6:**
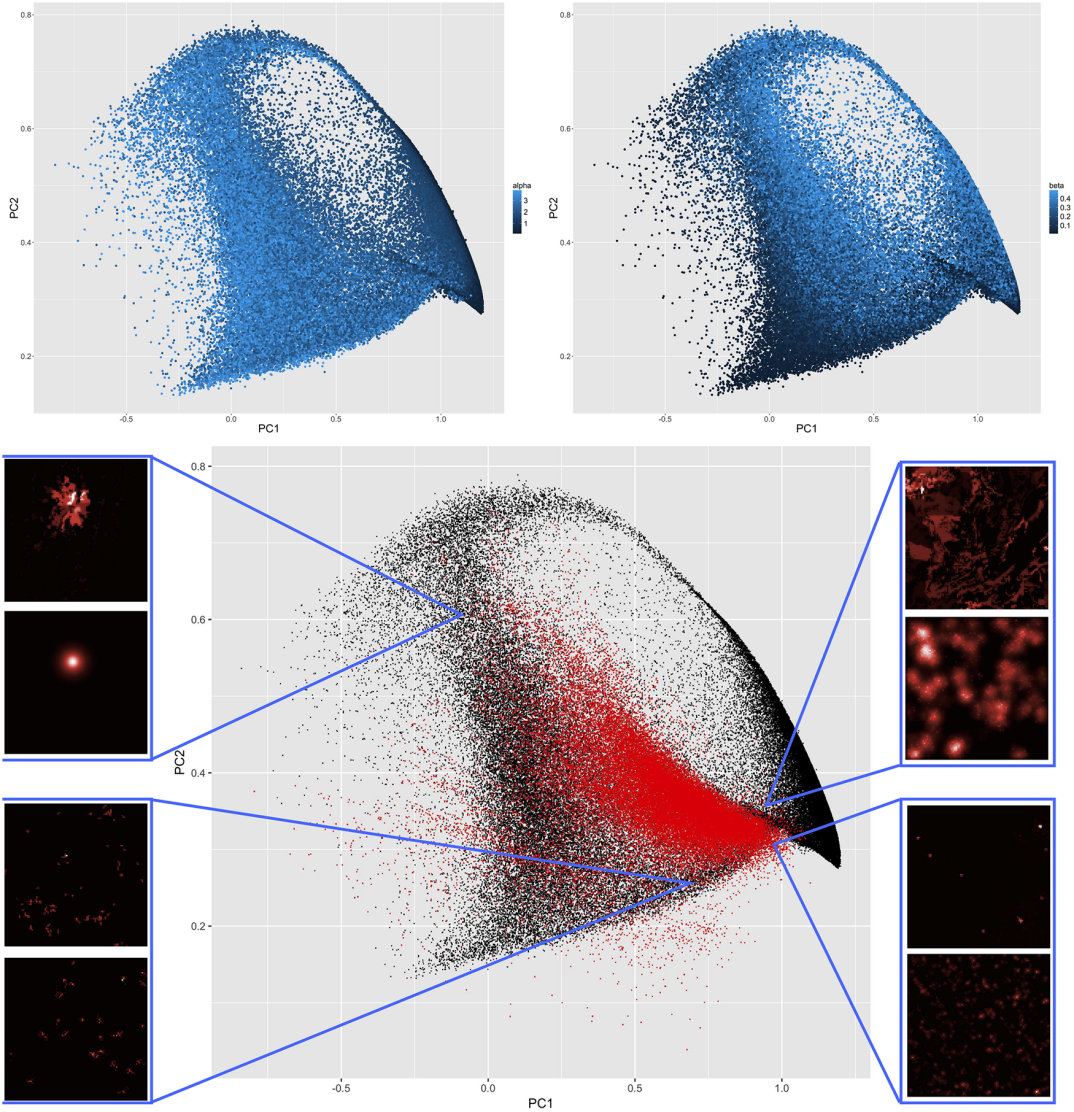
Model calibration. *(Top)* Simulated configurations in the two first principal components plan, color level giving the influence of *α* (left) and of *β* (right); *(Bottom)* Simulated points in the same space (in black) with real configurations (in red). We show around the plot typical examples of real configurations and their simulated counterparts in different regions of the space, the first being the real and the second the simulated in each case: Top left geographical coordinates 25.7361,44.69989—Romania, Bucharest—simulation parameters *α* = 3.87, *β* = 0.432, *N*_*G*_ = 1273, *nd* = 4, *P*_*m*_ = 63024; Top right geographical coordinates -2.561874,41.30203—Spain, Castilla et Leon, Soria—simulation parameters *α* = 1, *β* = 0.166, *N*_*G*_ = 100, *nd* = 1, *P*_*m*_ = 10017; Bottom left geographical coordinates 27.16068,65.889—Finland, Lapland—simulation parameters *α* = 0.4, *β* = 0.006, *N*_*G*_ = 25, *nd* = 1, *P*_*m*_ = 849; Bottom right geographical coordinates -2.607152,39.74274—Spain, Castilla-La Mancha, Cuenca—simulation parameters *α* = 1.14, *β* = 0.108, *N*_*G*_ = 637, *nd* = 1, *P*_*m*_ = 13235.

We consider a looser calibration constraint, by proceeding to a principal component analysis on synthetic and real morphological values. We then consider the two first components only, which represent 85% of cumulated variance. The rotated point clouds along these dimensions are shown in Fig 6. Most of the real point cloud falls in the simulated one in this simplified setting. We illustrate particular points with real configurations and their simulated counterparts: for example Bucharest, Romania, corresponds to a monocentric semi-stationary configuration, with very high aggregation but also diffusion and a rather low growth rate. Other examples show less populated areas in Spain and Finland.

From the subplots giving parameter influence, we show that most of real situations fall in the region with intermediate *α* but quite varying *β*. It is consistent with real scaling urban exponents which have a rather narrow variation range (between 0.8 and 1.3 generally [47]) compared to the one we allowed in the simulations, whereas the diffusion processes may be much more diverse.

This way, we have shown that the model is able to reproduce most of existing urban density configurations in Europe, despite its rather simplicity. It confirms that in terms of urban form, most of drivers at this scale can be translated into these abstract processes of aggregation and diffusion, but also that function must be quite correlated with form since the functional dimension (with an additional economic dimension in form for example) is not taken into account in the model.

## Discussion

### Main findings

We recall that our main findings are (i) an extensive knowledge of a simple aggregation-diffusion model of urban growth; and (ii) its calibration for a significant part of existing European urban forms. This study is to therefore a new attempt to empirically use this type of model which has been known for long but was mostly used as a toy-model as in [23]. Our contribution is also important as we illustrate the possibility to study the growth of urban form on large urban systems with a fine resolution, whereas previous studies spanned at most on a metropolitan area, such as the numerous applications of the Sleuth model [15]. The study of aggregation-diffusion processes at a continental scales opens avenues for more integrated policies and a switch from national urban growth management such as described by [48] to European-level policies, with an evidence-based relevance allowed by the application of such models at a large scale.

### Calibration and model refinement

Further work on this simple model may consist in extracting the exact parameter space covering all real situations and provide interpretation of its shape, in particular through the study of correlations between parameters and expressions of the boundaries functions. Its volume in different directions should furthermore give the relative importance of parameters.

**Fig 7:**
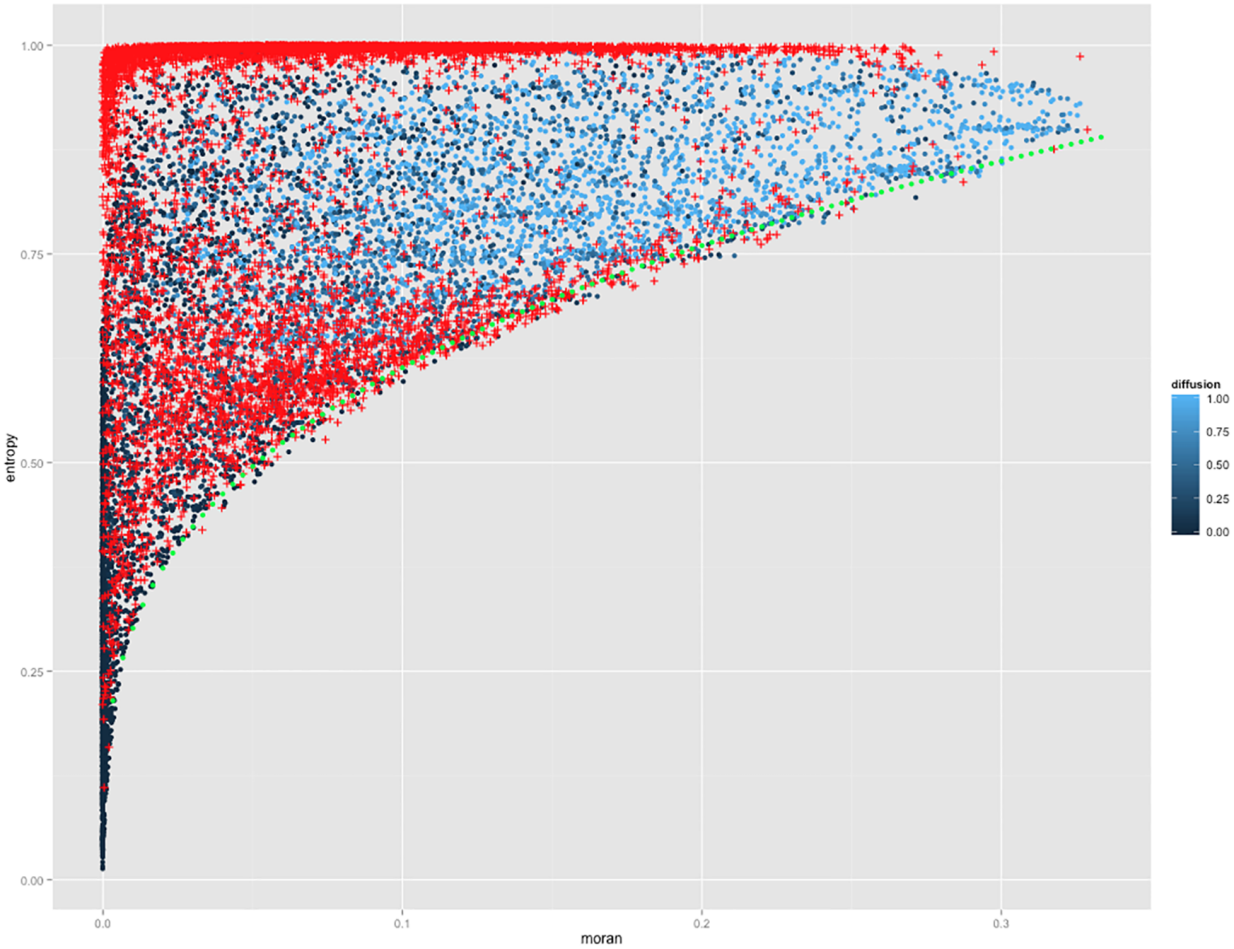
Exploration of the pattern space. Scatterplots of Moran against Entropy, with blue points obtained with LHS and red with the PSE algorithm exploration. Green dashed line gives the feasible lower bound.

Concerning the feasible space for the model of simulation itself, we tested a targeted exploration algorithm, giving promising results. More precisely, the pattern space exploration (PSE) algorithm [49] which is implemented in OpenMole, aims at determining all the possible outputs of a simulation models, i.e. samples its output space rather than input space. We obtain promising results as shown in Fig 7: we find that the lower bound in Moran-entropy plan, confirmed by the algorithm, unexpectedly exhibit a scaling relationship. This would imply that at a given level of auto-correlation, that one could want to attain for sustainability reasons for example (optimality through co-location), imposes a minimal disorder in the configuration of activities. Other relations between indicators and as a function of parameters can be the object of similar future developments.

The question of proceeding to a dynamical calibration of the model, i.e. trying to reproduce configurations at successive times, is conditioned to the availability of population data at this resolution in time.

We aimed at using abstract processes rather than having a highly realistic model. Tuning some mechanisms is possible to have a model closer to reality in microscopic processes: for example thresholding the local population density, or stopping the diffusion at a given distance from the center if it is well defined. It is however far from clear if these would produce such a variety of forms and could be calibrated in a similar way, as being accurate locally does not mean being accurate at the mesoscopic level for morphological indicators. Allowing the parameters to locally vary, i.e. being non stationary in space, or adding randomness to the diffusion process, are also potential model refinements.

### Integration into a multi-scale growth model

The question of the generic character of the model is also open: would it work as well when trying to reproduce urban forms on very different urban systems such as the United States or China. A first interesting development would be to test it on these systems and at slightly different scales (1km cell for example).

Finally, we believe that a significant insight into the non-stationarity of urban systems would be allowed by its integration into a multi-scale growth model. Urban growth patterns have been empirically shown to exhibit a multi-scale behavior [50]. Here at the mesoscopic scale, total population and growth rates are fixed by exogenous conditions of processes occurring at the macroscopic scale. It is indeed the aim of spatial growth models such as the Favaro-Pumain model [6] to determine such parameters through relations between cities as agents, the same way [51] indirectly infers information on interaction processes such as the tunnel effect. One would condition the morphological development in each area to the values of the parameters determined at the level above. In that setting, one must be careful of the role of the bottom-up feedback: would the emerging urban form influence the macroscopic behavior in its turn? Such multi-scale complex models are promising but must be considered carefully.

## Conclusion

In conclusion, we have provided a calibrated spatial urban morphogenesis model at the mesoscopic scale that can reproduce a consequent proportion of European urban patterns in terms of urban morphology. We show that the abstract processes of aggregation and diffusion are sufficient to capture urban growth processes at this scale. It is meaningful in terms of policies based on urban form such as energy efficiency, but also implies that issues out of the purely morphological scope must be tackled at other scales or through other dimensions of urban systems.

## Supporting information

S1 Text Extended model exploration. Extended figures for model exploration.
(PDF)

S2 Text Semi-analytical analysis. Analytical and numerical developments for the simplified model.
(PDF)

S3 Text Real indicators sensitivity. Sensitivity analysis of real indicators values to window size.
(PDF)
