# Supplementary material for: Calibration of a density-based model of urban morphogenesis
Source: PLoS One. 2018 Sep 6;13(9):e0203516. doi: 10.1371/journal.pone.0203516 (PMC6126859; doi:10.1371/journal.pone.0203516)
Supplement: S1 Text — Extended figures for model exploration. (PDF) [file pone.0203516.s001.pdf]

S1 Text : Extended Figures for Model Exploration

Convergence

Histograms for the 81 parameters points for which we did 100 repetitions are given in Fig. 1, for Moran index and slope indicators. Other indicators showed similar convergence patterns. The visual exploration of histograms confirms the numerical analysis done in main text for statistical convergence.

Indicators Behavior

We show in Fig.2 to Fig.5 the full behavior of all indicators, with all parameters varying, obtained through the extensive exploration, from which the plots in main text have been extracted. Because of the complex nature of emergent urban form, one can not predict output values without referring to this “exhaustive” parameter sweep.

Indicators Scatterplots

We show finally the full scatterplots of indicators, with real data points, in Fig. 6. These are preliminary step of the calibration on principal components, and we can see on these on which dimensions the model fails relatively to fit real data (in particular average distance).

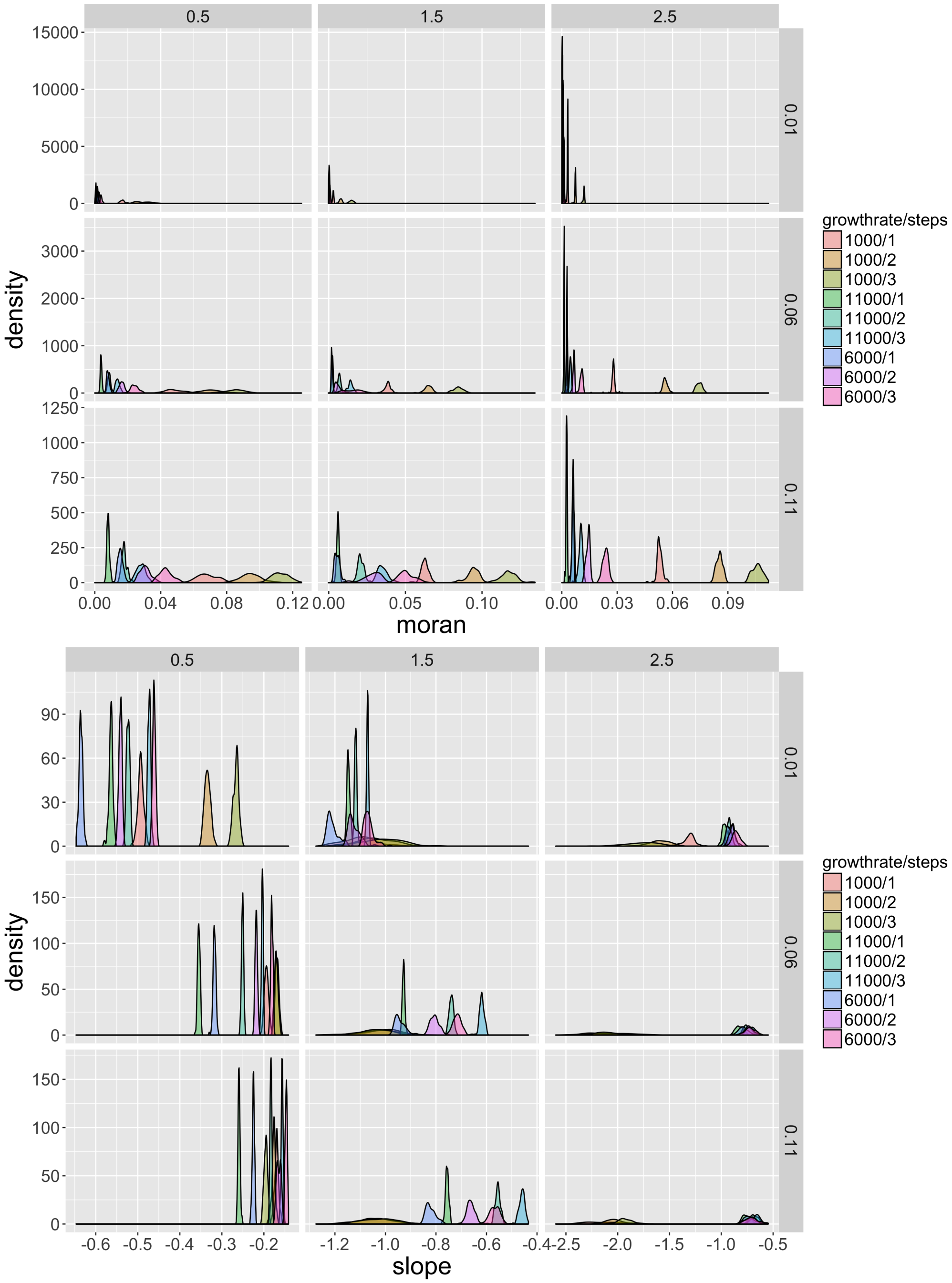

Figure 1. Histograms for Moran index (top) and slope (bottom), for varying α (columns), β (rows), N_G and n_d (colors).

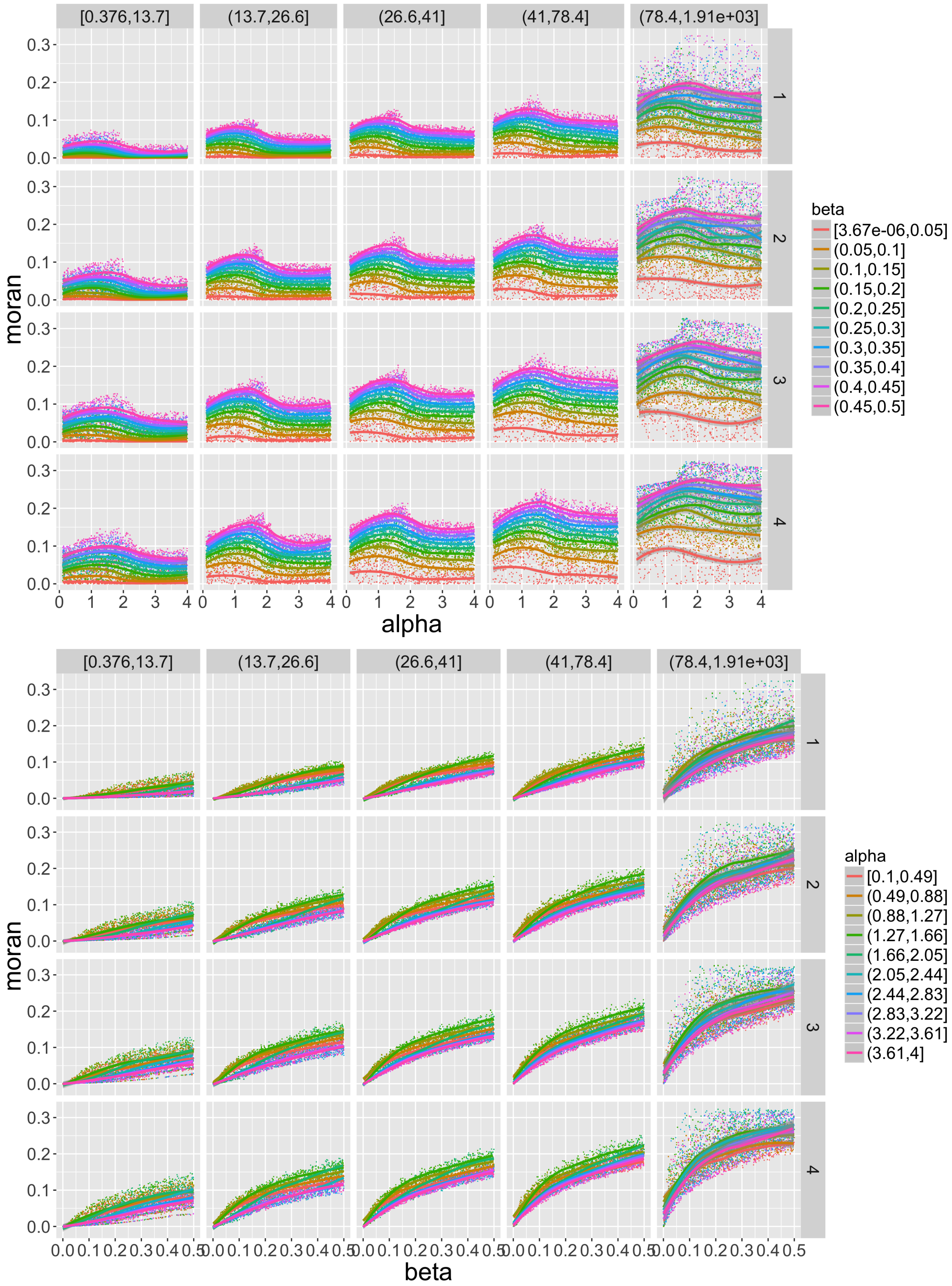

Figure 2. Moran index as a function of α (Top) and β (Bottom) for varying β (resp. α) given by color, and varying n_d (rows) and N_G (columns).

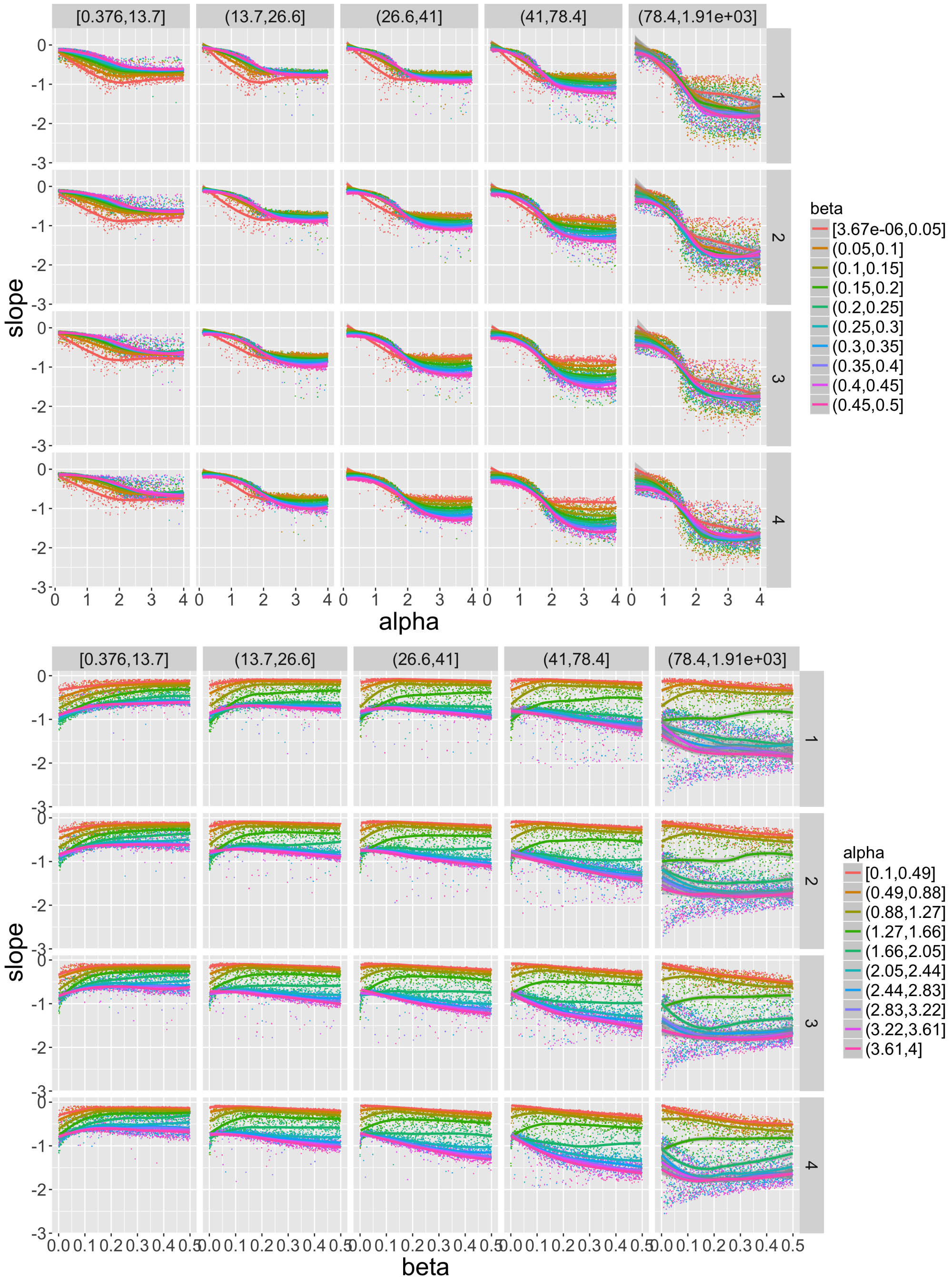

Figure 3. Slope as a function of α (Top) and β (Bottom) for varying β (resp. α) given by color, and varying n_d (rows) and N_G (columns).

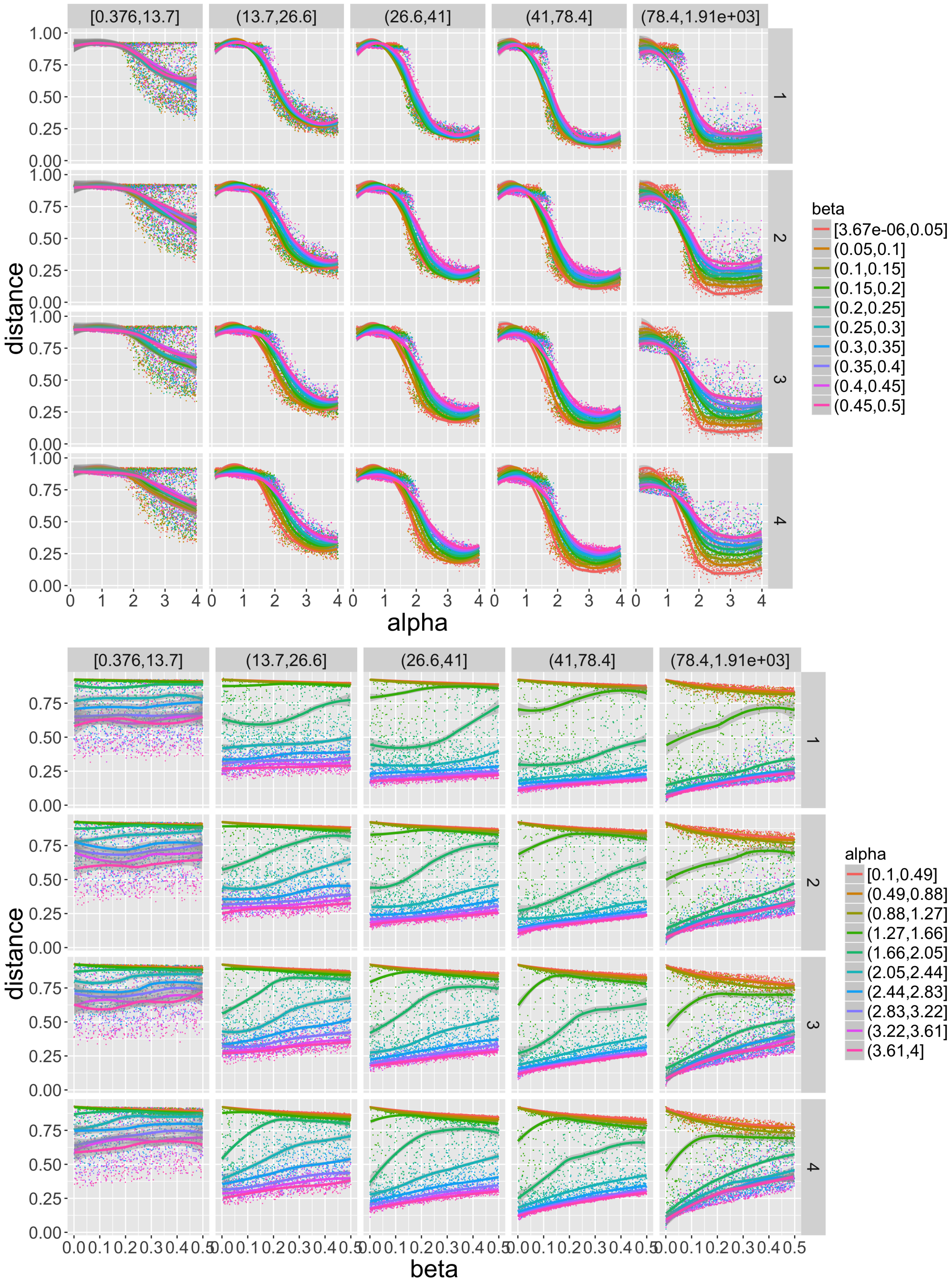

Figure 4. Average distance index as a function of α (Top) and β (Bottom) for varying β (resp. α) given by color, and varying n_d (rows) and N_G (columns).

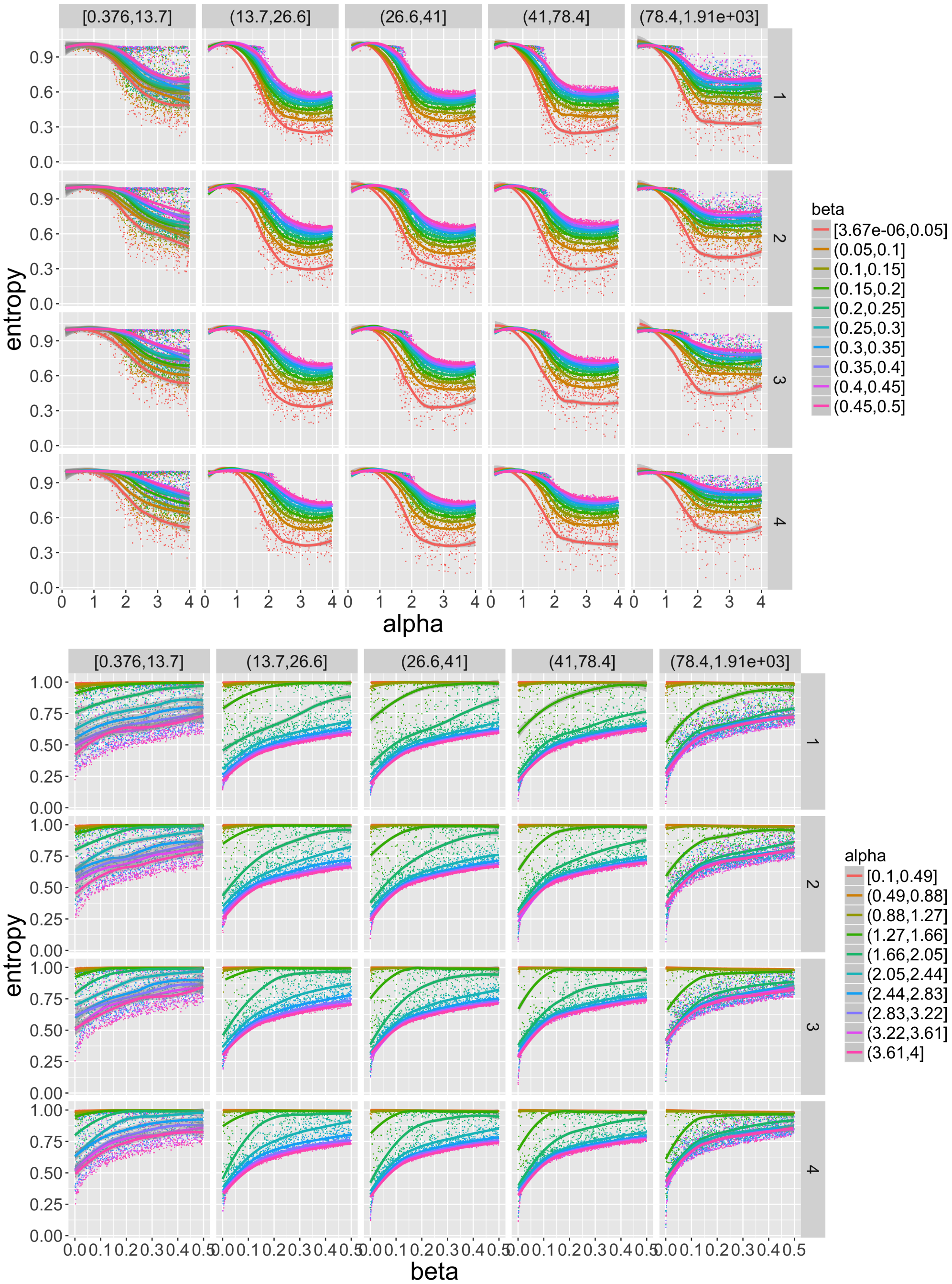

Figure 5. Entropy as a function of α (Top) and β (Bottom) for varying β (resp. α) given by color, and varying n_d (rows) and N_G (columns).

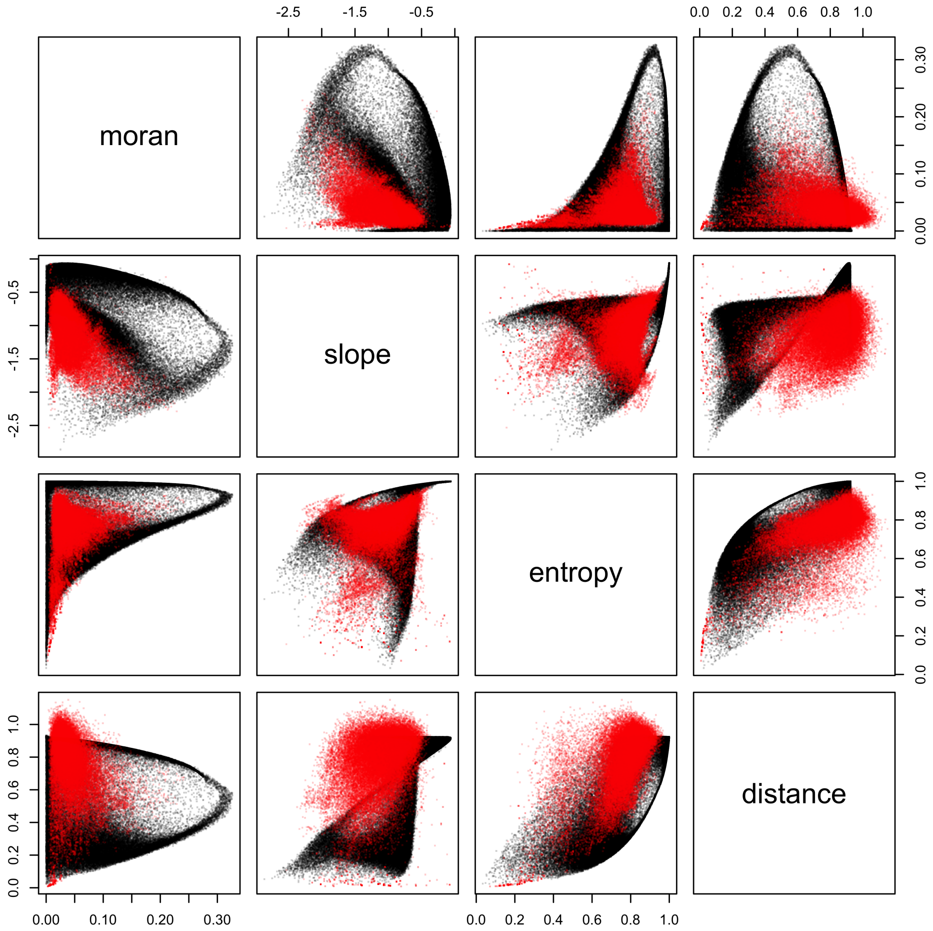

Figure 6. Scatterplots of indicators distribution in the sampled hypercube of the parameter space. Red points correspond to real data.
