# Supplementary material for: Calibration of a density-based model of urban morphogenesis
Source: PLoS One. 2018 Sep 6;13(9):e0203516. doi: 10.1371/journal.pone.0203516 (PMC6126859; doi:10.1371/journal.pone.0203516)
Supplement: S2 Text — Analytical and numerical developments for the simplified model. (PDF) [file pone.0203516.s002.pdf]

S2 Text : Semi-analytical analysis of the simplified model

Partial Differential Equation

We propose to derive the PDE in a simplified setting. To recall the configuration given in main text, the system has one dimension, such that $x \in \mathbb{R}$ with $1/\delta x$ cells of size δx, and we use the expected values of cell population $p(x,t) = \mathbb{E}[P(x,t)]$. We furthermore take $n_d = 1$. Larger values would imply derivatives at an order higher than 2 but the following results on the existence of a stationary solution should still hold.

Denoting $\tilde{p}(x,t)$ the intermediate populations obtained after the aggregation stage, we have

$$\tilde{p}(x,t) = p(x,t) + N_g \cdot \frac{p(x,t)^\alpha}{\sum_x p(x,t)^\alpha}$$

since all populations units are added independently. If $\delta x \ll 1$ then $\sum_x p^\alpha \simeq \int_x p(x,t)^\alpha dx$ and we write this quantity $P_\alpha(t)$. We furthermore write $p = p(x,t)$ and $\tilde{p} = \tilde{p}(x,t)$ in the following for readability.

The diffusion step is then deterministic, and for any cell not on the border ($0 < x < 1$), if δt is the interval between two time steps, we have

$$\begin{aligned} p(x, t+\delta t) &= (1-\beta) \cdot \tilde{p} + \frac{\beta}{2} \left[\tilde{p}(x-\delta x, t) + \tilde{p}(x+\delta x, t) \right] \\ &= \tilde{p} + \frac{\beta}{2} \left[(\tilde{p}(x+\delta x,t) - \tilde{p}) - (\tilde{p} - \tilde{p}(x-\delta x, t)) \right] \end{aligned}$$

Assuming the partial derivatives exist, and as $\delta x \ll 1$, we make the approximation $\tilde{p}(x+\delta x, t) - \tilde{p} \simeq \delta x \cdot \frac{\partial \tilde{p}}{\partial x}(x,t)$, what gives

$$(\tilde{p}(x+\delta x,t) - \tilde{p}) - (\tilde{p} - \tilde{p}(x-\delta x,t)) = \delta x \cdot \left(\frac{\partial \tilde{p}}{\partial x}(x,t) - \frac{\partial \tilde{p}}{\partial x}(x-\delta x, t) \right)$$

and therefore at the second order

$$p(x,t+\delta t) = \tilde{p} + \frac{\beta \delta x^2}{2} \cdot \frac{\partial^2 \tilde{p}}{\partial x^2}$$

Substituting $\tilde{p}$ gives

$$\begin{aligned}\frac{\partial^2 \tilde{p}}{\partial x^2} &= \frac{\partial^2 p}{\partial x^2} + \frac{N_G}{P_\alpha} \cdot \frac{\partial}{\partial x}\left[\alpha \frac{\partial p}{\partial x} p^{\alpha-1}\right] \\ &= \frac{\partial^2 p}{\partial x^2} + \alpha \frac{N_G}{P_\alpha}\left[\frac{\partial^2 p}{\partial x^2} p^{\alpha-1} + (\alpha-1)\left(\frac{\partial p}{\partial x}\right)^2 p^{\alpha-2}\right]\end{aligned}$$

By supposing that $\frac{\partial p}{\partial t}$ exists and that δt is small, we have $p(x,t+\delta t) - p(x,t) \simeq \delta t \frac{\partial p}{\partial t}$, what finally yields , by combining the results above, the partial differential equation

$$\delta t \cdot \frac{\partial p}{\partial t} = \frac{N_G \cdot p^\alpha}{P_\alpha(t)} + \frac{\alpha\beta(\alpha-1)\delta x^2}{2} \cdot \frac{N_G \cdot p^{\alpha-2}}{P_\alpha(t)} \cdot \left(\frac{\partial p}{\partial x}\right)^2 + \frac{\beta \delta x^2}{2} \cdot \frac{\partial^2 p}{\partial x^2} \cdot \left[1 + \alpha \frac{N_G p^{\alpha-1}}{P_{\alpha(t)}}\right] \quad (1)$$

Initial conditions should be specified as $p_0(x) = p(x,t_0)$. To have a well-posed problem similar to more classical PDE problems, we need to assume a domain and boundary conditions. A finite support is expressed by $p(x,t) = 0$ for all t and x such that $|x| > x_m$.

Stationary solution for density

The non-linearity and the integral terms making the equation above out of the scope for analytical resolution, we study its behavior numerically in some cases. Taking a simple initial condition $p_0(0) = 1$ and $p_0(x) = 0$ for $x \neq 0$, we show that on a finite domain, density $d(x,t)$ always converge to a stationary solution for large t, for a large set of values of (α, β) with fixed $N_G = 10$ ($\alpha \in [0.4, 1.5]$ varying with step 0.025 and $\log \beta \in [-1, -0.5]$ with step 0.1). We show in Fig. 1 the corresponding trajectories on a typical subset. The variation of the asymptotic distribution as a function of α and β are not directly visible, as they depend on very low values of the outward flows at boundaries. We give in Fig. 2 their behavior, by showing the value of the maximum of the distribution. Low values of β give an inversion in the effect of α, whereas high values

of β give comparable values for all α.

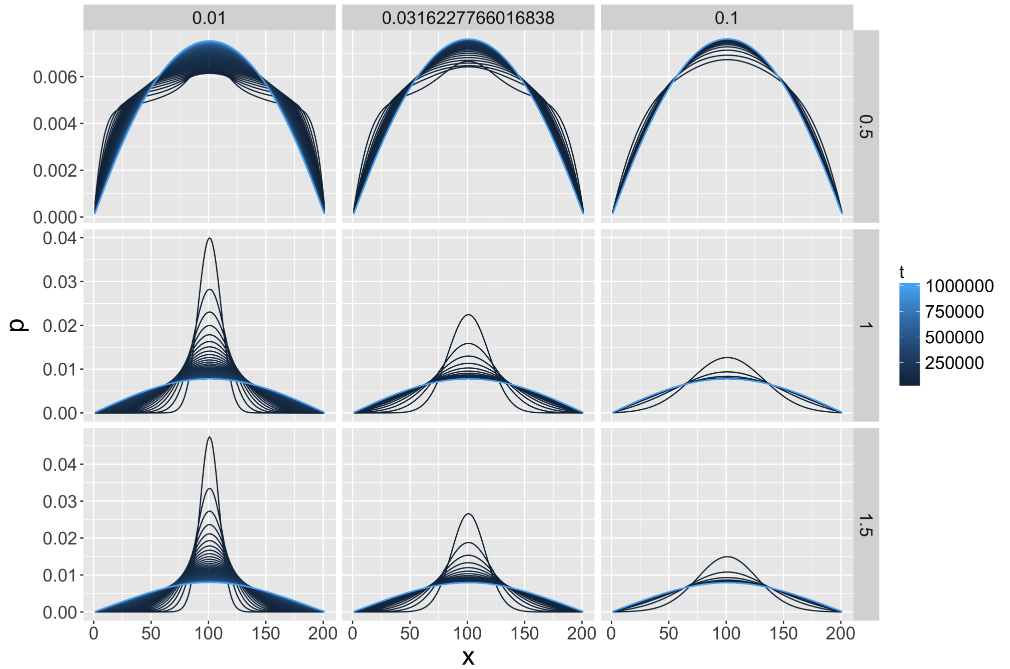

Figure 1. Trajectories of densities as a function of the spatial dimension, for varying β (columns) and α (rows). Color gives time.

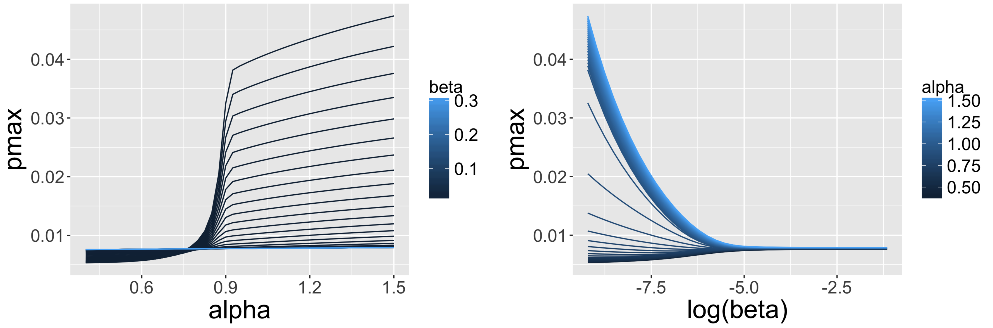

Figure 2. Dependency of $\max d(t \to \infty)$ to α and β.
