# Supplementary material for: Calibration of a density-based model of urban morphogenesis
Source: PLoS One. 2018 Sep 6;13(9):e0203516. doi: 10.1371/journal.pone.0203516 (PMC6126859; doi:10.1371/journal.pone.0203516)
Supplement: S3 Text — Sensitivity analysis of real indicators values to window size. (PDF) [file pone.0203516.s003.pdf]

S3 Text : Sensitivity of indicators to spatial resolution

We evaluate here the sensitivity of morphological indicators to grid size. We show in Fig. 1 morphological indicators, mapped for France, for different grid sizes. The sizes taken here, in correspondance to the 50km scale used in main results, are at similar magnitudes: we test windows of size 30km and 100km. The offsets are in each case half of the window (15km and 50km respectively). It is possible to see with eyeball validation that some indicators have a low sensitivity, the change in scale resembling a smoothing of the finer field: for example for morphology in the case of Moran, entropy and hierarchy. Average distance, which is indeed rather noisy at the smaller scale, is necessarily sensitive to aggregation, what is consistent with a sensitivity expected at smoothing.

This comparison, on the one hand is to be taken with caution because of the difficulty to directly compare scales for indicators, and on the other hand stays limited. We propose then a method to quantify the variability of indicators to window size. Let X_D and X_d two spatial fields corresponding to two spatial scales $D > d$ (that we take as characteristic distances). The fields are assumed discrete at points respectively denoted by $\left(\vec{x}_i^{(D)}\right)_{1\leq i\leq N_D}$ and $\left(\vec{x}_j^{(d)}\right)_{1\leq i\leq N_d}$. The idea is to compare a smoothing of the finer field to the field with the larger scale: if the correlation between these two values is high, it is possible to deduce one field from the other by aggregation and the scale of computation does not influence final results in an other way than the final resolution. Let $W_{ij} = \left(\exp -d_{ij}/d_0\right)_{ij}$ a matrix of spatial weights computed with euclidian distances d_{ij} between the points $\vec{x}_i^{(D)}$ and $\vec{x}_j^{(d)}$. Then with $W'_{ij} = W_{ij}/\sum_j W_{ij}$, we can compute the spatial smoothing of X_d at the points $\vec{x}_i^{(D)}$, with the matrix product

$$\tilde{X}_d(\vec{x}_i^{(D)}) = W' * \vec{x}_j^{(d)}$$

The correlation is then given by $\rho\left[\tilde{X}_d, X_D\right]$ estimated on all $\vec{x}_i^{(D)}$ points.

The Fig. 2 gives the variation of this correlation for all (D, d) couples, with a variable

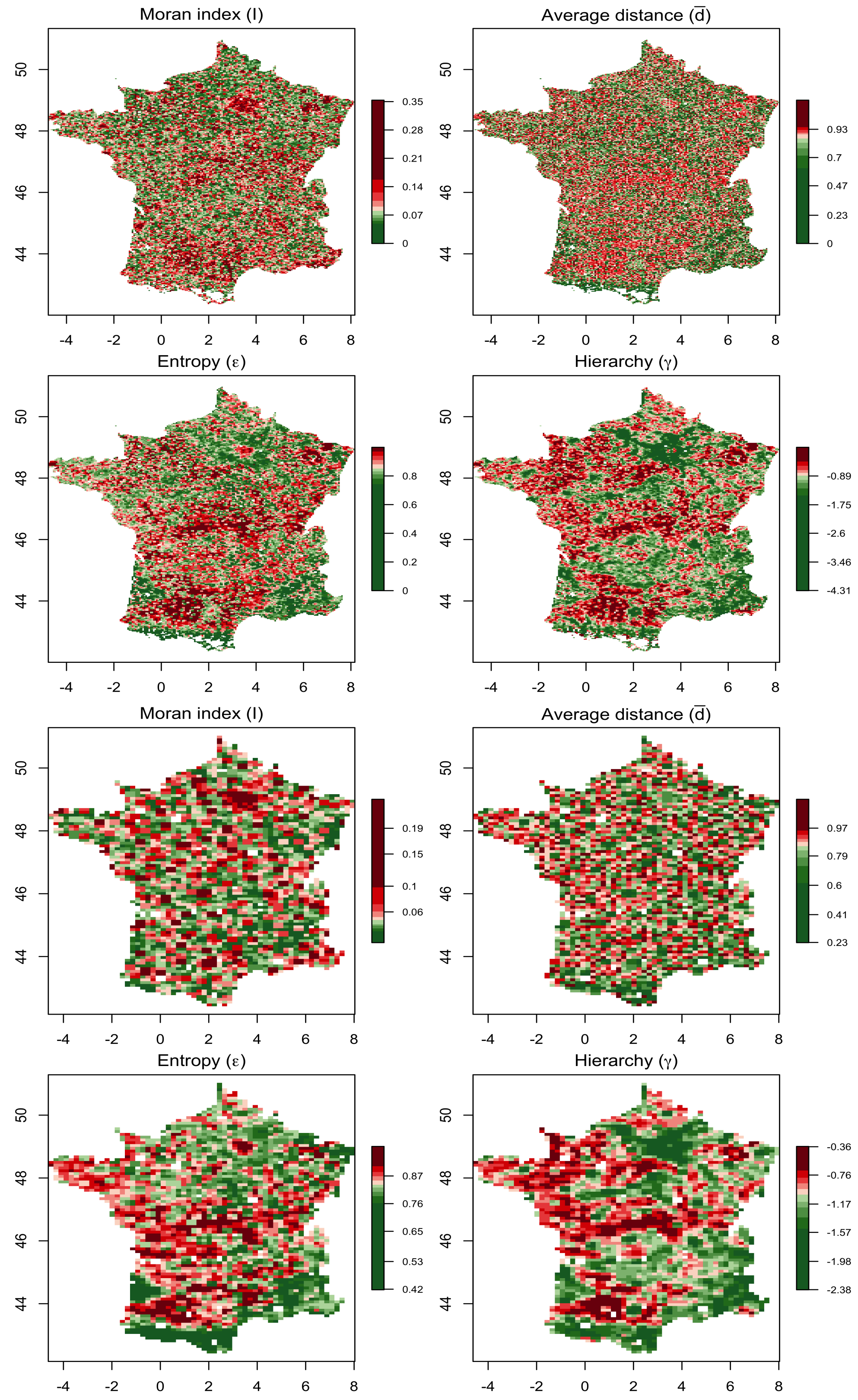

Figure 1. Morphological indicators for different grid sizes. The first four maps show the indicators computed on a window of size 30km, the last four maps with a window of size 100km.

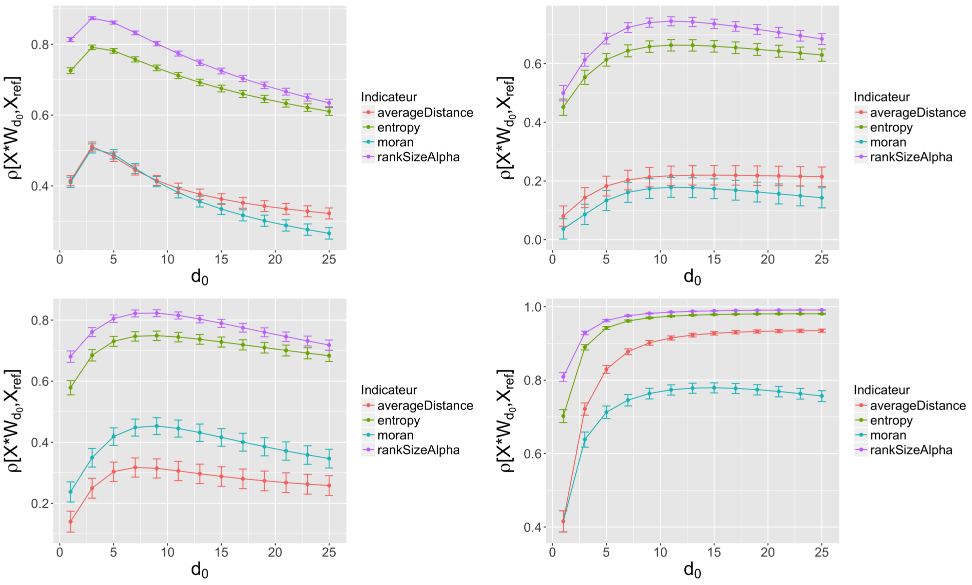

Figure 2. Correlations between indicators computed at different scales. From left to right and top to bottom, $(d = 30, D = 50)$, $(d = 30, D = 100)$, $(d = 50, D = 100)$, and the last plot gives the correlation between the two fields $d_1 = 30$ and $d_2 = 50$ both smoothed at the characteristic distance of d_0.

d_0 for smoothing. We generally observe the existence of a maximum, which corresponds to the optimal smoothing level to deduce the larger scale from the finer. The largest correlations on all indicators are obtained for $D = 50$km and $d = 30$km, what means that indicators are not very sensitive to small variations in small window sizes. As expected, the lowest correlations are obtained for the largest scale difference (100/30km). Morphological indicators have the same qualitative behavior across combinations, and we find the behavior suggested by the previous maps (entropy and hierarchy being the less sensitive, Moran index and average distance a bit more sensitive). For all indicators, the sensitivity remains however reasonable. Finally, a smoothing of both fields yields asymptotic maximal correlations with very high values: the computation window size does not matter if we consider smoothed fields.
